# Enrichment from Birth Accelerates the Functional and Cellular Development of a Motor Control Area in the Mouse

**DOI:** 10.1371/journal.pone.0006780

**Published:** 2009-08-26

**Authors:** Teresa Simonetti, Hyunchul Lee, Michael Bourke, Catherine A. Leamey, Atomu Sawatari

**Affiliations:** Discipline of Physiology, School of Medical Sciences and the Bosch Institute, University of Sydney, Sydney, Australia; L'université Pierre et Marie Curie, France

## Abstract

**Background:**

There is strong evidence that sensory experience in early life has a profound influence on the development of sensory circuits. Very little is known, however, about the role of experience in the early development of striatal networks which regulate both motor and cognitive function. To address this, we have investigated the influence of early environmental enrichment on motor development.

**Methodology/Principal Findings:**

Mice were raised in standard or enriched housing from birth. For animals assessed as adults, half of the mice had their rearing condition reversed at weaning to enable the examination of the effects of pre- versus post-weaning enrichment. We found that exclusively pre-weaning enrichment significantly improved performance on the Morris water maze compared to non-enriched mice. The effects of early enrichment on the emergence of motor programs were assessed by performing behavioural tests at postnatal day 10. Enriched mice traversed a significantly larger region of the test arena in an open-field test and had improved swimming ability compared to non-enriched cohorts. A potential cellular correlate of these changes was investigated using Wisteria-floribunda agglutinin (WFA) staining to mark chondroitin-sulfate proteoglycans (CSPGs). We found that the previously reported transition of CSPG staining from striosome-associated clouds to matrix-associated perineuronal nets (PNNs) is accelerated in enriched mice.

**Conclusions/Significance:**

This is the first demonstration that the early emergence of exploratory as well as coordinated movement is sensitive to experience. These behavioural changes are correlated with an acceleration of the emergence of striatal PNNs suggesting that they may consolidate the neural circuits underlying these behaviours. Finally, we confirm that pre-weaning experience can lead to life long changes in the learning ability of mice.

## Introduction

Both genetic and environmental factors can profoundly affect the ontogeny and execution of complex behaviours. Enrichment paradigms, which provide increased opportunities for sensory stimulation, motor activity and social interaction [1], [2], have been instrumental in revealing the influence of an animal's surroundings on neural development and function. Environmental enrichment can elicit widespread changes in an animal's behavioural and cognitive abilities [3], reducing anxiety and increasing exploratory behaviour [4]–[7], as well as improving the acquisition of learning tasks [8]–[13]. Early exposure to enriched conditions can lead to increases in brain weight, cortical thickness [14]–[17], dendritic branching, synaptic density [18]–[22], and changes in the regulation of neural plasticity [23]. In the visual system, the onset of the critical period for sensitivity to altered visual experience occurs earlier in enriched mice [24], and the precocious closing of this developmental epoch is associated with an acceleration in the appearance of perineuronal nets (PNNs). PNNs are composed of chondroitin sulphate proteoglycans (CSPGs) [25], extracellular matrix molecules associated with the consolidation of inhibitory circuits [25]–[27]. The maturation of visual acuity is also accelerated in animals raised in enriched environments [25]–[28].

Although the influence of enrichment on sensory and learning systems has garnered much interest, less is known about how enrichment affects the maturation of circuits underlying motor control and function. Since the onset of self-initiated coordinated movements occurs early in the postnatal period [29]–[31], the consolidation of circuits regulating motor control may be especially sensitive to early enrichment. Cortico-striatal networks play an integral part in the initiation of volitional behaviour. The neostriatum (caudate/putamen), in particular, has been implicated in playing a vital role in regulating motivational drive and coordinated movement. Histologically defined striatal subregions [32], [33] subserve different, if related, functions: mu-opioid receptor (μOR1) positive striosomes are involved in regulating reward-based signalling, while the matrix plays key roles in sensory-motor processing including the control and maintenance of motor behaviour [34], [35]. We have recently shown that CSPG expression in the mouse striatum is highly dynamic during the first two postnatal weeks with diffuse, striosome-associated CSPG-positive ‘clouds’ (present at postnatal day (P)4) beginning to give way to PNN expression in the matrix at around P10; PNN density approaches adult levels by P14 [36]. By analogy with the visual system [37], the rapid appearance of striatal PNNs suggests that they may demarcate a form of ‘critical period’ for the formation and consolidation of striatal circuits. Although previous studies have shown that striatal receptor expression is sensitive to experience during a brief period around one month postnatal [38]–[40], virtually nothing is known about how experience may influence the initial consolidation of these circuits within the first two to three weeks after birth when motor programs are emerging.

As a first step, we have examined whether environmental enrichment from birth yields long-term behavioural changes in adult mice. We find that enrichment limited to just the first three postnatal weeks improves adult acquisition of a spatial learning task to the same extent as life-long enrichment. We also examined the effect of enrichment on the ontogeny of motor behaviours and found that raising pups in enhanced environments significantly increases exploratory behaviour in open field tests and improves motor coordination while swimming at P10. Finally, to identify a potential cellular basis for these changes, we assessed the impact of enrichment on the development of CSPG expression and found that the compartmental transition within the striatum is also accelerated. Our findings show that pre-weaning enrichment can profoundly affect early striatal development and may contribute to long-term, measurable changes in complex behaviour.

## Results

### Enrichment confined to the pre-weaning period improves adult performance on a spatial learning task

**Figure 1:**
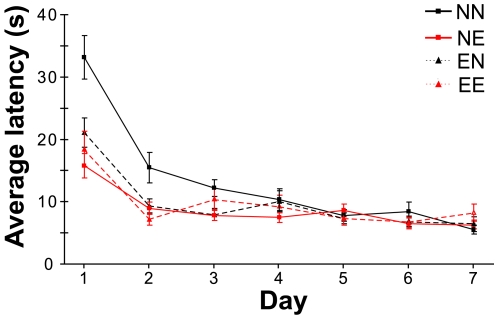
Enrichment limited to the pre-weaning period improves performance in the Morris water maze (MWM) to a level equivalent to life-long enrichment. Average MWM latencies across 7 training days of non-enriched mice (NN, solid black, n = 10), mice enriched from weaning (NE, solid red, n = 16), enriched from birth until weaning (EN, dashed black, n = 7) and enriched from birth into adulthood (EE, dashed red, n = 7). Mice receiving enrichment performed significantly better across the 7 training days relative to non-enriched mice (Mixed model ANOVA, F(3, 36) = 8.48, p<0.001; see text).

To confirm that early (pre-weaning) enrichment has long-term effects on learning ability, mice were raised in four different combinations of pre- and post-weaning environmental conditions (non-enriched, non-enriched, NN (n = 10); non-enriched, enriched, NE (n = 16); enriched, non-enriched, EN (n = 7); enriched, enriched, EE (n = 7)) to adulthood. At 8–12 weeks of age, performance in the Morris water maze (MWM) [41] was assessed over 7 days. We found that enrichment significantly affected acquisition of the spatial learning task (mixed model ANOVA, main effect of between subject comparison of groups, F(3, 36) = 8.48, p<0.001, Fig. 1). Specifically, mice that had experienced enrichment at any point during their lifetime – from weaning (NE), from birth (EE), or for just the first 3 weeks of life (EN) – had significantly lower latencies to complete the task compared to mice raised entirely in non-enriched, standard conditions (NN) (Dunnett's T3 test: NN vs. NE, p = 0.034; NN vs. EN, p = 0.039; NN vs. EE, p = 0.046). No significant difference was detected between the groups in the performance of a platform displaced probe (repeated measures ANOVA, between subject comparison of groups, F(3, 36) = 0.045, p = 0.987). These results suggest that even a brief period of early enrichment affects acquisition of the water-maze in adults. Moreover, the fact that latencies exhibited by the four groups converged by roughly the third day of training indicates that the initial differences are unlikely to be due to differences in swim rates or their ability to detect sensory cues.

### Early enrichment affects ‘exploratory’ behaviour in pre-weaned postnatal mice

**Figure 2:**
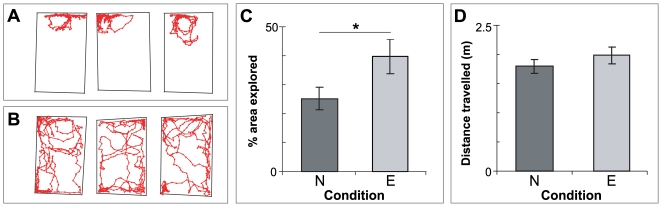
Enrichment increases exploratory behaviour in an ‘open field’ test in young mice. Representative traces (red) of (A) non-enriched and (B) enriched P10 mice. Enriched mice typically traverse a much higher proportion of the test arena than non-enriched mice. (C) Percentage of area explored for non-enriched (‘N’, n = 17) and enriched (‘E’, n = 17) P10 mice, as obtained from the traces. The increase in the percentage of the open-field explored by enriched mice is statistically significant (t-test, p = 0.0237). *: p<0.03. (D) Total cumulative distance travelled by non-enriched and enriched mice. There was no detectable difference in absolute distance travelled between the two groups (Student's t-test, p = 0.334).

Although early environmental enrichment has been shown to influence performance of adult animals on a number of cognitive and non-cognitive tasks [3], [5], [9], the role of enrichment on the ontogeny of behaviour is less clear. Complex tasks such as the MWM often require the coordinated activation of a number of neural circuits including those involved in perception, motivation, decision making, and initiation of action. The maturation of these circuits varies in both onset and duration [42], [43]. Enrichment has been shown to affect the development of sensory cortex, influencing the maturation as well as functionality of circuits required for the perception of the environment [26], [27]. It is not known whether enrichment would similarly affect the development of circuits underlying motor and exploratory behaviour in mice. It is conceivable that motor pathways could be more “hard-wired” and thus less sensitive to early experiences than sensory circuits. Two major histochemically-defined compartments of the striatum, the striosomes and the matrix, contribute differentially to functional striatal networks: striosomes are part of the neural pathways associated with reward and motivation, while cells in matrix are linked to circuits involved in sensory-motor control and function [34], [35]. The pathways linked to these striatal compartments have different developmental trajectories [44], [45]. Accordingly, the influence of enrichment on the behaviours dependent on the maturation of these circuits may be most distinguishable when they first emerge. To address this possibility, we recorded the movement trajectories of P10 mice raised from birth in either enriched or non-enriched environments in a simple open-field enclosure (see Methods). Representative examples of trajectories of non-enriched and enriched pups are given in figures 2A and 2B. We observed that enriched pups traversed a significantly greater proportion of the test arena compared to non-enriched pups (Enriched (n = 17) area explored: 40±6.95%; Non-enriched (n = 17): 25±4.62%; Student's t-test: p = 0.02365; Fig. 2C). The absolute distance travelled, however, was not significantly different between the two groups (Enriched distance travelled: 1982±147.195 mm; Non-enriched: 1796±119.32 mm; Student's t-test: p = 0.334; Fig. 2D). These data are consistent with an increased capacity for directed movement and/or exploratory drive in enriched pups.

### Early enrichment affects motor performance in juvenile mice

**Figure 3:**
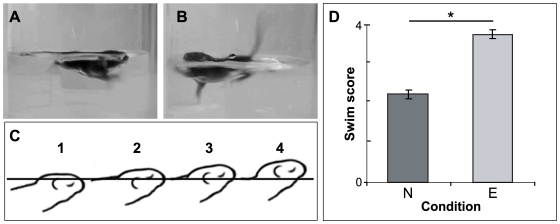
Pre-weaning enrichment improves swimming performance at P10. (A, B) Representative examples of swimming behaviour of non-enriched (A) and enriched (B) mice at P10. In non-enriched mice (A), only the crown of the head and dorsal tip of the nose are above the water level (score of 2). In enriched (B) mice, the bulk of the head, including the entire ear and nose are above the water level (score of 4). The back and tail are also notably more elevated. (C) Drawings illustrating the relative position of the eyes, nose and ears used in the scoring assessment (based on the scheme of St Omer [47]). (D) Graph plotting swim scores for non-enriched (‘N’, n = 17) versus enriched (‘E’, n = 17) P10 mice. Scores were made by an observer blind to treatment group. Enriched mice scored significantly higher than non-enriched controls (Student's t-test, p<0.001). *: p<0.001.

Although open field behaviour revealed no changes in locomotor function (measured in terms of distance travelled) due to enrichment, it is possible that this measurement alone does not have the sensitivity to reveal differences in coordinated motor activity between the two groups at the age tested. Open-water swimming has been demonstrated to be a robust indicator of motor coordination in developing rodents [46], [47]: a pup's ability to keep its nose above water is highly correlated with the age and maturation of motor circuitry in juvenile animals. Moreover, swimming behaviour itself exhibits a rapid transition to a more mature state during the middle of the second postnatal week [48]–[50]. Accordingly, we compared the swimming ability of P10 mouse pups raised from birth in either enriched or non-enriched environments to determine if enrichment can influence the ontogeny of this coordinated motor activity. Images illustrating representative examples of swimming performance in non-enriched and enriched pups are given in figures 3A and 3B. When we rated swimming prowess (based on the pup's ability to elevate their nose and head above water; see Methods; Fig. 3C) using an observer blind to treatment conditions, we found that enriched mice performed markedly better than their non-enriched counterparts. These differences were significant (Enriched (n = 17) swim scores: 3.765±0.106; Non-enriched (n = 17): 2.235±0.106; Student's t-test: p<0.001; Fig. 3D). Thus, our open-field and swim test results together suggest that the onset and maturation of both exploratory and coordinated motor behaviours are accelerated in juvenile mice that have experienced enrichment from birth.

### Transition of striatal CSPG expression is accelerated in pups enriched from birth

We have recently revealed that CSPG expression exhibits a compartment-specific transition in the mouse striatum: diffuse, CSPG-positive clouds, almost exclusively associated with striosomes in the first postnatal week, start to dissipate, and by P10 PNNs begin appearing, predominantly within the matrix [36]. At this stage the density of PNNs is low, but this increases rapidly over the ensuing days [36]. The time course of the appearance of striatal PNNs overlaps with the period when rodent pups begin to walk consistently with all four limbs [29], [31], [51] and with the transition to a more adult-like swimming behaviour [48]–[50]. This raises the possibility that the precocious onset of exploratory as well as coordinated motor behaviours, observed here in enriched pups at P10, may be a result of accelerated maturation of motivational and sensory-motor circuits within the caudate/putamen. We therefore asked if changes in CSPG expression in the striatum may be a cellular correlate of the observed behavioural changes. To assess this possibility, we compared CSPG patterning in the developing striatum of enriched and non-enriched mouse pups at two age points (P8 and P10) corresponding to the period when striatal CSPG expression is just beginning to undergo the compartmental transition.

**Figure 4:**
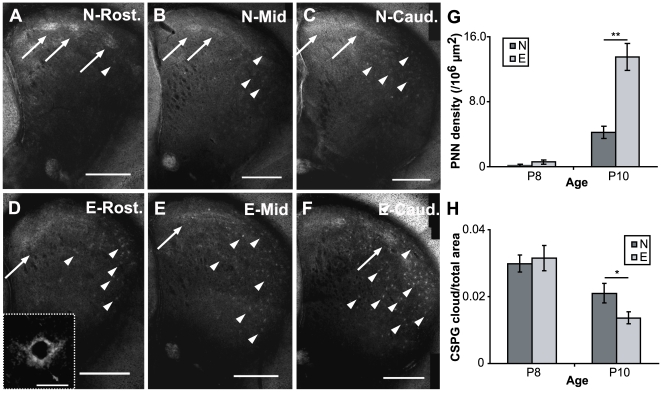
Enrichment accelerates the striatal maturation as determined by the pattern of CSPG staining at P10. (A–C) A rostral (rost.) to caudal (caud.) series showing the pattern of CSPG staining in the striatum at P10 in non-enriched mice. CSPG staining in the striatum is most prominent in diffuse clouds (some highlighted by arrows) which we have previously shown to be associated with striosomes [36]. PNNs, characterised by more punctuate staining, can also be seen in the matrix (arrowheads) but these are present at very low density. Only one or two can be seen in sections from rostral striatum (A), though they become slightly more numerous in mid (B) and caudal (C) sections. (D–F) A similar series to that shown in A–C but for enriched mice. Here, the CSPG-associated clouds (arrows) are much less apparent. PNNs are, however, markedly more numerous and more prominent across all rostrocaudal levels compared to non-enriched mice. The inset in 4D shows the appearance of the PNNs at higher power. Note the characteristic lattice-like network of CSPGs encircling the soma and proximal dendrites. Scalebars: 500 µm in A–F, 20 µm for the Inset in D. (G–H) Graphs plotting increases in PNN (G) and decreases in CSPG cloud (H) densities in enriched mice at P10. Differences are significant (see text for details). Nine sections from 3 animals were quantified for each group. *: p<0.05, **: p<0.001.

We found that the age-dependent increase in PNN formation within the matrix, as well as the decrease in striosomal CSPG cloud density, was accelerated in the striatum of enriched pups. As described previously, PNNs were first observed in standard-housed mice at P10. They were mostly present in the dorso-lateral striatum within the mid and caudal regions ([36], Fig. 4B,C, arrowheads). Very few PNNs were present in the more rostral sections (Fig. 4A) at this stage. In comparison, a much higher density of PNNs could be clearly observed in all striatal sections of enriched mice (Fig. 4D–F). In caudal sections, PNNs were more robust and present in higher numbers (Fig. 4F) than in equivalent sections from non-enriched mice (Fig. 4C); PNNs were also clearly detectable in rostral sections from enriched pups (Fig. 4D), unlike in non-enriched cohorts (Fig. 4A). A high power confocal image in the inset of figure 4D confirms that the appearance of these structures is similar to that previously described [36], [52]. We also investigated PNN expression at an earlier time point, P8. We found that there were very few PNNs present at this stage in either enriched or non-enriched mice (not shown).

Quantitative analysis across these two developmental time points confirmed that striatal PNN density increased significantly with age for both groups (Multi-factorial ANOVA main effect of age, F(1, 50) = 43.983, p<0.001; Fig. 4G). This confirms and extends our previous findings in mice raised in standard conditions [36]. Enrichment also profoundly affected PNN formation, with enriched pups exhibiting significantly higher densities compared to non-enriched cohorts (Multi-factorial ANOVA main effect for enrichment, F(1, 50) = 14.390; p<0.001; Fig. 4G). The significant interaction between the two factors (Interaction, F(1, 50) = 11.926, p = 0.001) indicated that environmental enrichment accelerated the formation of these proteoglycan structures soon after they were first detected in the developing striatum (Fig. 4G). The fact that PNN densities rapidly changed from being effectively identical at P8 (t-test, p = 0.138), to exhibiting significant differences at P10 (t-test, p<0.001) between the two groups, reinforces the notion that enrichment affects the rate, not the onset, of PNN formation.

CSPG clouds, previously shown to be associated with μOR1 positive striosomes [36], were also less pronounced in the striatum of enriched mice (Fig. 4D–F, arrows) compared to their non-enriched counterparts at P10 (Fig. 4A–C, arrows). When we compared these expression levels to CSPG cluster levels at P8, we found that age also had an effect on the expression of these structures: significant decreases in CSPG cloud density were observed in both enriched and non-enriched pups between P8 and P10 (Multi-factorial ANOVA main effect of age, F(1, 50) = 20.665, p<0.001; fig. 4H). Enrichment conditions, however, did not have a significant effect on CSPG expression when compared across ages (Multi-factorial ANOVA main effect for enrichment, F(1, 50) = 0.955; p = 0.333). A moderate interaction (Interaction, F(1, 50) = 2.307, p = 0.135), indicated that enrichment may influence the developmentally regulated decrease in CSPG densities. Similar to PNN formation, CSPG clouds showed little difference between the two groups at P8 (t-test, p = 0.734; Fig. 4H). By P10, however, the measured density of CSPG clusters was significantly lower in enriched pups (t-test, p = 0.043; Fig. 4H). Although not nearly as dramatic, the developmental changes previously observed in CSPG clouds densities, much like those of PNNs, are accelerated in mice raised in enriched environments. Together, these changes are consistent with the hypothesis that enrichment accelerates the maturation of both motivational and sensory-motor circuits within the striatum.

## Discussion

This study confirms that enrichment during the early postnatal period can dramatically affect measurable behaviour throughout the life of an animal. Mice raised in an enriched environment for only the first three postnatal weeks acquired a spatial navigation task (MWM) at a rate similar to that of animals enriched their entire lives, and faster than those which had never been enriched. We also demonstrated that enrichment can influence the ontogeny of both exploratory and motor behaviour. When exposed to an open-field, juvenile (P10) enriched mice explored a significantly greater area of the environment compared to non-enriched counterparts. The development of coordinated movement was also affected by enrichment, with enriched pups exhibiting superior swimming skills relative to non-enriched cohorts of the same age. Finally, we found that the transition in CSPG expression in the striatum, a potential marker for the maturation of circuitry underlying both exploratory and motor behaviours, is also significantly accelerated in enriched mice. Together, these results have important implications for the role of environmental influences on early postnatal motor development and the maturation of striatal circuits.

### Early enrichment can influence adult acquisition of a spatial learning task

Previous studies have demonstrated that early exposure to enriched environments can affect sensory function [26], [27], untrained behaviours (e.g., grooming, rearing) [53], [54], as well as performance on tests that examine anxiety levels [6], [55], [56] and cognitive function [3], [9], [57]. Our current data reveal that pups enriched exclusively for the first three postnatal weeks exhibit adult MWM acquisition rates similar to those of mice enriched for their entire lives. This observation is consistent with previous work in rats [3] demonstrating that early experience has a life-long impact on performance of complex learning tasks.

While ostensibly a test of spatial learning and memory, performance on the MWM can be influenced by a number of other factors, including procedural learning capacities [58]–[60] as well as anxiety levels [57], [61]. When considering the rate of adult MWM acquisition between enriched and non-enriched cohorts, dramatic differences were observed only during the first few days of training. Since early enrichment has the potential to affect emotive, sensory, as well as cognitive systems [26], [27], [57], it is difficult to determine which neural circuits most directly contributed to the observed differences in MWM performance. Further experiments will be required to reveal which factors affected by early enrichment most influence adult task acquisition. Nevertheless, the demonstration that pre-weaning enrichment can affect life-long learning ability reinforces the importance of early postnatal experiences on cognitive development.

### Enrichment from birth can affect exploratory behaviour in early postnatal mice

Assessing the behaviour of any freely moving animal is difficult due to an inability to isolate the specific sensory cues and motivational states the subjects utilise to acquire or perform a task. This is even more challenging when considering developing animals, as the emergence and refinement of coordinated actions contribute to the overall complexity of the assessed behaviour. In rodents, locomotor activity emerges rapidly in early development [51], with pups engaging in a period of forelimb dominated ambulation [29], [30] before the onset of adult-like walking (i.e., the coordinated use of all four limbs) early in the second postnatal week (P8 for mice [30]). This is consistent with our finding that the distance travelled by enriched and non-enriched P10 groups did not differ when exposed to open-fields. Our observations did reveal, however, that enriched mice ‘explored’ a greater area of the open–field compared to their non-enriched counterparts.

Exploratory behaviour has been linked to both the maturation and activation of reward related circuitry. Nest-related olfactory cues, as well as systemic injections of L-DOPA, a precursor to the reward signal dopamine, can induce early expression of locomotor behaviour in neonatal rodents [62], [63]. Moreover, mice lacking the dopamine D4 receptor, one of three D2-like receptors expressed in cortical and subcortical regions associated with planning and motivation [64], [65], exhibit decreased exploratory behaviour [35]. Increased motivational levels as a result of the precocious development of reward circuits due to enrichment may therefore contribute to the increased exploration observed in the enriched mouse pups.

The influence of environmental enrichment on the development of the visual system is well established [26], [27]. Although the pups in this study were tested before eye opening, the possibility that early maturation of other sensory systems may have contributed to the observed differences in enriched pups cannot be ruled out at this stage. Moreover, anxiety, cortisol, and neurotrophin levels are also known to be affected by environmental factors in adults [66]–[68]. It remains possible that enrichment from birth may similarly influence the stress response of neonatal mice, either directly or through the mother [66], [69], contributing to the changes observed in open field performance. Further experiments will be required to determine to what degree the accelerated maturation of reward circuits, as well as pathways involved in regulating sensory and emotive responses, influences exploratory behaviour in enriched early postnatal mice.

### Early enrichment can influence swimming performance in early postnatal mice

The development of coordinated motor activity was revealed to be accelerated in enriched P10 mice. When challenged with an open swim task, enriched pups exhibited superior performance compared to their non-enriched counterparts. Swimming is an ethologically relevant response which requires the integration of a number of basic sensory-motor programs [49]. It has therefore been employed in the past as a means of assessing the ontogeny of coordinated motion. Although a thorough assessment of limb kinematics was beyond the scope of this study, previous work has demonstrated that both limb movement patterning and coordination involved in swimming change during early postnatal development [49], [50]. It is possible that an accelerated maturation of circuits regulating motor coordination, due to enrichment, is contributing to the differences in performance observed in enriched neonates. Swimming behaviour has the potential to be influenced by differences in maturation rates of circuitry underlying motivation, anxiety, and sensory processing [49]. Further experiments, however, will be required to discern the contribution of all these factors to the differences in swimming behaviour observed between enriched and non-enriched pups.

### Accelerated transition of striatal CSPG structures in enriched pups

Although a multitude of sensory and non-sensory modalities may contribute to the differences in both exploratory and motor behaviour due to early enrichment, one intriguing mechanism underlying the discrepancy is a potential difference in the maturation of motivational circuits between enriched and non-enriched pups. In light of this possibility, it is of particular interest that enriched pups exhibit an accelerated transition of striatal CSPG expression compared to non-enriched counterparts of the same age. To the best of our knowledge, this is the first concrete example of a measureable, early postnatal (within the first three postnatal weeks) anatomical change due to environmental enrichment in a mammalian motor control pathway. Recent work has shown that CSPG-associated PNNs begin forming in the mouse caudate/putamen by P10, reaching adult-like densities by P14 [36], the approximate age range when a host of sensory and motor changes occur, including eye opening [26], as well as the emergence of walking as the predominant means of locomotion by juvenile rodents [29], [31], [51]. PNNs are known to be associated with parvalbumin-positive fast-spiking interneurons in cortical and subcortical areas [52], [70]. In the striatum, fast-spiking parvalbumin positive neurons receive direct cortical and nigrostriatal input [71]–[73]. The formation of striatal PNNs may thus reflect a consolidation of these connections, akin to increased PNN expression demarcating the closing of the visual cortical ‘critical period’ due to monocular deprivation [37]. Moreover, the accelerated compartmental transition of striatal CSPG expression we observed in enriched animals shares some intriguing similarities with changes observed in visual cortical PNN expression in mice raised in non-standard environments from birth [24]. It will be of interest to determine whether the accelerated appearance of striatal PNNs is the cause or consequence of paralleled acceleration of motor ability. The fact that digestion of PNNs in visual cortex can re-open the critical period for monocular deprivation [37] suggests that it may indeed be causal. It would also be of interest to determine whether the expression of other cellular correlates of inhibitory interneuron maturation, such as brain derived nerve growth factor (BDNF), Otx2, and insulin-like growth factor 1 (IGF-1) are also accelerated in the striatum [25], [74], [75] in a manner similar to that reported in visual cortex [76].

### Maternal and environmental factors contributing to neonatal enrichment

In our hands, enrichment from birth yielded measureable changes indicative of a precocious development of motor control and motivational circuits. It should be noted, however, that in our enrichment protocol, we did not differentiate maternal rearing effects from direct environmental factors as potential influences on the developing mice. Numerous studies have demonstrated that habitat can affect maternal behaviour [26], [77], which in turn influence both neural function and behaviour of offspring [23], [78]. It is possible that changes in maternal rearing due to enrichment of the mothers alone could lead to at least some of the dramatic differences in striatal maturation rates and motor/exploratory performance we observed here in enriched pups.

The enriched cages were designed to provide enhanced stimulation of all sensory modalities available to the neonatal pups. Although visual features within the environment would presumably have limited effect on pups until eye opening (P12 for enriched mice [26]), other sensory modalities, particularly the olfactory system, can influence neonatal development from a much younger age. Indeed, the presentation of olfactory cues has been shown to induce coordinated motor behaviour of rat pups as young as P3 [62], considerably earlier than when similar movements can be observed in free-moving conditions. Although further experiments will be required to determine how specific features of our enriched environments contribute to the change observed to both circuitry and function in enriched pups, our results provide evidence that enrichment from birth can profoundly influence the maturation of motor control systems in developing animals.

### Multiple critical periods across striatal development

Previous work has shown that the rat neostriatum, a vital motor control structure, exhibits a late developmental ‘critical’ period (between P30 and P37), during which the expression of both muscarinic acetylcholine receptors [38], [39], as well as dopamine D2 receptors [38], [40] can be altered due to experience. This epoch, however, is considerably later than the period during which many of the behaviours associated with this important motor structure have begun to emerge. The transition in CSPG expression described in this study occurs at a time consistent with the initial onset of striatum dependent behaviours, such as the initiation and maintenance of goal directed, coordinated movements. Work performed in the visual cortex has indicated the presence of multiple critical periods [79], [80], and it is possible that the striatum also exhibits disparate, temporally defined periods of increased sensitivity to environmental factors. Further studies will be required to determine how the marked transition in striatal CSPG expression early in life, and the period of receptor expression plasticity within the caudate/putamen later in development are related. Nevertheless, the fact that both this striatal CSPG transition and behaviours associated with striatal function exhibit accelerated development due to enrichment indicates that there exists a period that is crucially important for the maturation of motor control circuits within the first two to three postnatal weeks of the neonatal mouse.

In the wild, mice experience a varied and dynamic environment, full of challenges, in the form of avoiding predators, as well as the need to continuously seek sustenance. Although enriched environments by no means pose anywhere near the same level of threat to the animal, they do provide a greater proximity to natural experiences compared to standard housing conditions. Given that enrichment can reverse cellular, as well as behavioural, deficits in mice missing key long-term potentiation intermediaries [23], [78], delay the onset of symptomology in Huntington's and Parkinson's mouse models [81], [82], and influence both in the short-term as well as life-long performance on tasks designed to assess cognitive function [3], [9], it is of vital interest to determine how and which neural circuits are affected by ‘improving’ an animal's environment. Our results provide evidence that enrichment from birth can accelerate the maturation of motor control and motivational circuits, at the cellular as well as the behavioural level, akin to changes that have been described in sensory as well as memory systems [23], [26], [27], [77]. These changes correlate with long-term improvements in the acquisition of learning tasks following early-enrichment. The demonstration that the development of this key brain area is profoundly sensitive to the environment in the first weeks of life highlights the critical role which experience plays during this important developmental epoch. The fact this is also associated with life-long improvements in learning underscores its importance and has implications for early childhood learning and intervention programs.

## Methods

### Ethics statement

All procedures were approved by the Animal Ethics Committee of the University of Sydney and conformed to National Health and Medical Research Council of Australia guidelines. Procedures were performed on C57/BL6 mice which were reared at the University of Sydney animal house facility. All mice were housed in a single adequately-ventilated room in 21°C ambient temperature on a 12-hour light-dark cycle with ad libitum access to dry food and water.

### Housing of animals in standard and enriched environments

On arrival in the colony, half of the pregnant dams were assigned to standard housing (30 cm×13 cm×13 cm cage), and the other half to enriched housing (45 cm×30 cm×13 cm cage). The standard environment contained a red mouse igloo and some added tissues for bedding material. The enriched environment contained many additional objects (a running wheel, plastic tubing, rubber balls, scented plush balls, a bell-ball, Velcro strips and high-contrast visual stimuli). Items were moved to different locations within each cage every 2–3 days. Two enriched litters were housed together to provide additional opportunities for social interaction.

For adult mice, four environmental conditions were established, based on the order of exposure to the standard and enriched environment in the pre- and post-weaning periods. Mice assigned to the non-enriched non-enriched (NN) group received no enrichment. Mice assigned to the non-enriched enriched (NE) group received enrichment from weaning age (P21) to adulthood. Mice assigned to the enriched non-enriched (EN) group received enrichment until P21. Mice assigned to the enriched enriched (EE) condition received enrichment for their entire lives.

### Behavioural testing in adult mice: Morris water maze

Adult male mice (8–12 weeks of age) from all four conditions (NN (n = 10), NE (n = 16), EN (n = 7) and EE (n = 7); all groups sourced from at least 3 litters each) underwent Morris water maze [41] training for 7 consecutive days. On the 8^th^ day, the four groups also submitted to a displaced, submerged platform probe. Each day consisted of four trials, and each trial began with a randomly-selected starting position at the outside edge of one of four quadrants; with each quadrant used once as a starting position. The trial began when the mouse was gently lowered by the tail into the water at the starting position. The trial ended when the mouse reached the platform and remained there for 10 s. The latency to reach the platform from the starting position was measured. Any trial that went for longer than 60 s was terminated and the mouse was placed on the platform for 10 s. In this case, the latency to reach the platform was recorded as 60 s. The mouse was then removed from the platform and returned to its home cage. For the 7 day acquisition, a mixed model ANOVA (with group as the between-subject factor, and days as the within-subject repeated measure) combined with a Dunnett's T3 posthoc test was used to reveal performance differences between the groups (p<0.05 defining significance). A repeated measures ANOVA (group as between-subject factor, trial as within-subject repeated measure) was used to assess group differences in probe behaviour.

### Open field testing in early postnatal mice

P10 mice from standard (n = 17) and enriched (n = 17) conditions were subject to an ‘open field’ test. Pups were sourced from 4 separate litters for each group. Prior to and during testing, mice were kept in their home cages in a waiting room adjacent to the testing room. Each pup was removed from its home cage and placed in a small lidless box (12 cm×20 cm) made of translucent white plastic, and behaviour was digitally recorded (Logitech QuickCam digital video camera) for 2 min. At the end of the trial, the pup was returned to its home cage. Each clip was decompiled at 3 Hz (to yield 360 frames). In each frame, the tip of the animal's nose was manually marked, and the co-ordinates of all 360 nose-tip positions were plotted in series to form a trace of the animal's movements around the box. The percentage of area explored was obtained by expressing the area of the trace outline as a percentage of the total area of the test enclosure. Differences between the groups were assessed using a Student's t-test, with p<0.05 defining significance.

### Forced swimming in early postnatal mice

Enriched (n = 17) and non-enriched (n = 17) P10 pups, sourced from 4 litters each, were exposed to a forced swimming test. Prior to and during testing, mice were kept in their home cages in a waiting room adjacent to the testing room. At the start of the trial, the mouse was gently lowered by the tail into the centre of the ‘pool’ (a 1 litre beaker filled to 600 ml with 31°C tap water). The experimenter then left the room. The mouse was allowed to swim for 10 s, after which it was removed from the pool and returned to its home cage. All trials were digitally recorded as above. The video recordings were used to give each mouse an overall score (ranging from 0 to 4) depending on the position of its nose and ears with respect to the surface of the water [47]. A score of 1 was assigned if the dorsal part of the nose was level with or below the water surface, 2 if the nose was at or above the surface but the ears were still below, 3 if the nose was above the surface and the ears were level with the surface, and 4 if both the nose and ears were above the surface (a schematic diagram illustrating the scale used for scoring is given in Fig. 3C). Differences in values between the groups were assessed using a Student's t-test, with p<0.05 defining significance.

### Measuring PNN density and CSPG cloud areas in early postnatal mice

P8 and P10 mice from standard and enriched conditions (3 mice per group per age for a total of 12 mice) were euthanised with >100 mg/kg of sodium pentobarbitone i.p., then transcardially perfused with 0.9% saline followed by 4% paraformaldehyde in 0.1 M phosphate buffer (PB). Brains were extracted, post-fixed overnight, and cryoprotected in 30% sucrose in PB. Brains were then embedded in gelatin-albumin hardened with glutaraldehyde and sectioned coronally at 60 µm on a freezing microtome. 3 sections per brain, from the rostral pole of the striatum to the crossing of the anterior commissure, were selected for analysis. These sections were labelled for CSPGs using Wisteria floribunda agglutinin (WFA), a plant lectin used to visualise CSPGs, as described previously [36]. Sections were mounted onto glass slides and air dried overnight. The slides were dehydrated in increasing concentrations of ethanol, defatted in histolene and coverslipped using Entellan mountant. Sections were digitally imaged using Zeiss deconvolution microscope with AxioCam HR camera for low-power images, Zeiss LSM 510 META confocal microscope for high-power images. In each imaged slice, PNNs were manually marked and cross-sectional area of CSPG clouds, as well as the entire cross-sectional striatal area, were manually outlined (Neurolucida), and both CSPG cloud and PNN density measurements were obtained. Multi-factorial ANOVAs (enrichment condition and age as factors) were used to compare CSPG cloud and PNN expression densities across groups and developmental time points. Student's t-tests (p<0.05) were used to compare density values between enrichment condition groups at each age point.

## References

[1] van Praag H, Kempermann G, Gage FH (2000). Neural consequences of environmental enrichment.. Nature Reviews Neuroscience.

[2] Krech D, Rosenzweig MR, Bennett EL (1962). Relations between brain chemistry and problem-solving among rats raised in enriched and impoverished environments.. Journal of Comparative & Physiological Psychology.

[3] Hebb DO (1947). The effects of early experience on problem-solving at maturity.. American Psychologist.

[4] Iwata E, Kikusui T, Takeuchi Y, Mori Y (2007). Fostering and environmental enrichment ameliorate anxious behavior induced by early weaning in Balb/c mice.. Physiology & Behavior.

[5] Widman DR, Rosellini RA (1990). Restricted daily exposure to environmental enrichment increases the diversity of exploration.. Physiology & Behavior.

[6] Larsson F, Winblad B, Mohammed AH (2002). Psychological stress and environmental adaptation in enriched vs. impoverished housed rats.. Pharmacology Biochemistry and Behavior.

[7] Swanson HH, McConnell P, Uylings HBM, Van Oyen HG, Van de Poll NE (1983). Interaction between pre-weaning undernutrition and post-weaning environmental enrichment on somatic development and behaviour in male and female rats.. Behavioural Processes.

[8] Harburger LL, Lambert TJ, Frick KM (2007). Age-dependent effects of environmental enrichment on spatial reference memory in male mice.. Behavioural Brain Research.

[9] Iso H, Simoda S, Matsuyama T (2007). Environmental change during postnatal development alters behaviour, cognitions and neurogenesis of mice.. Behavioural Brain Research.

[10] Frick KM, Fernandez SM (2003). Enrichment enhances spatial memory and increases synaptophysin levels in aged female mice.. Neurobiology of Aging.

[11] Williams BM, Luo Y, Ward C, Redd K, Gibson R (2001). Environmental enrichment: Effects on spatial memory and hippocampal CREB immunoreactivity.. Physiology & Behavior.

[12] Pietropaolo S, Feldon J, Alleva E, Cirulli F, Yee BK (2006). The role of voluntary exercise in enriched rearing: a behavioral analysis.. Behavioural Neuroscience.

[13] Wainwright PE, Lévesque S, Krempulec L, Bulman-Fleming B, McCutcheon D (1993). Effects of environmental enrichment on cortical depth and morris-maze performance in B6D2F2 mice exposed prenatally to ethanol.. Neurotoxicology and Teratology.

[14] Krech D, Rosenzweig MR, Bennett EL (1960). Effects of environmental complexity and training on brain chemistry.. Journal of Comparative & Physiological Psychology.

[15] Bennett EL, Diamond MC, Krech D, Rosenzweig MR (1964). Chemical and anatomical plasticity of brain.. Science.

[16] Rosenzweig MR, Krech D, Bennett EL, Diamond MC (1962). Effects of environmental complexity and training on brain chemistry and anatomy: a replication and extension.. Journal of Comparative & Physiological Psychology.

[17] Cummins RA, Livesy PJ, Bell JA (1982). Cortical depth changes in enriched and isolated mice.. Developmental Psychobiology.

[18] Greenough WT, Volkmar FR (1973). Pattern of dendritic branching in occipital cortex of rats reared in complex environments.. Experimental Neurology.

[19] Globus A, Rosenzweig MR, Bennett EL, Diamond MC (1973). Effects of differential experience on dendritic spine counts in rat cerebral cortex.. Journal of Comparative & Physiological Psychology.

[20] Uylings HBM, Kuypers K, Diamond MC, Veltman WAM (1978). Effects of differential environments on plasticity of dendrites of cortical pyramidal neurons in adult rats.. Experimental Neurology.

[21] Holloway JRL (1966). Dendritic branching: some preliminary results of training and complexity in rat visual cortex.. Brain Research.

[22] Faherty CJ, Kerley D, Smeyne RJ (2003). A Golgi-Cox morphological analysis of neuronal changes induced by environmental enrichment.. Developmental Brain Research.

[23] Li S, Tian X, Hartley DM, Feig LA (2006). The environment versus genetics in controlling the contribution of MAP kinases to synaptic plasticity.. Curr Biol.

[24] Bartoletti A, Medini P, Berardi N, Maffei L (2004). Environmental enrichment prevents effects of dark-rearing in the rat visual cortex.. Nature Neuroscience.

[25] Ciucci F, Putignano E, Baroncelli L, Landi S, Berardi N (2007). Insulin-like growth factor 1 (IGF-1) mediates the effects of enriched environment (EE) on visual cortical development.. PLoS ONE.

[26] Cancedda L, Putignano E, Sale A, Viegi A, Berardi N (2004). Acceleration of visual system development by environmental enrichment.. Journal of Neuroscience.

[27] Sale A, Putignano E, Cancedda L, Landi S, Cirulli F (2004). Enriched environment and acceleration of visual system development.. Neuropharmacology.

[28] Prusky GT, Reidel C, Douglas RM (2000). Environmental enrichment from birth enhances visual acuity but not place learning in mice.. Behavioural Brain Research.

[29] Altman J, Sudarshan K (1975). Postnatal development of locomotion in the laboratory rat.. Anim Behav.

[30] Fox WM (1965). Reflex-ontogeny and behavioural development of the mouse.. Anim Behav.

[31] Westerga J, Gramsbergen A (1990). The development of locomotion in the rat.. Brain Res Dev Brain Res.

[32] Chesselet MF, Graybiel AM (1983). Met-enkephalin-like and dynorphin-like immunoreactivities of the basal ganglia of the cat.. Life Sci.

[33] Graybiel AM, Chesselet MF (1984). Compartmental distribution of striatal cell bodies expressing [Met]enkephalin-like immunoreactivity.. Proc Natl Acad Sci U S A.

[34] Allen JP, Hathway GJ, Clarke NJ, Jowett MI, Topps S (2003). Somatostatin receptor 2 knockout/lacZ knockin mice show impaired motor coordination and reveal sites of somatostatin action within the striatum.. Eur J Neurosci.

[35] Dulawa SC, Grandy DK, Low MJ, Paulus MP, Geyer MA (1999). Dopamine D4 receptor-knock-out mice exhibit reduced exploration of novel stimuli.. J Neurosci.

[36] Lee H, Leamey C, Sawatari A (2008). Rapid reversal of chondroitin sulfate proteoglycan distribution in subcompartments of mouse neostriatum during the emergence of behaviour.. PLoS ONE.

[37] Pizzorusso T, Medini P, Berardi N, Chierzi S, Fawcett JW (2002). Reactivation of ocular dominance plasticity in the adult visual cortex.. Science.

[38] Ibarra GR, Paratcha GC, Wolansky MJ, Azcurra JM (1996). Co-alteration of dopamine D2 receptor and muscarinic acetylcholine receptor binding in rat striatum after circling training.. Neuroreport.

[39] Ibarra GR, Rodriguez JA, Paratcha GC, Azcurra JM (1995). Permanent alteration of muscarinic acetylcholine receptor binding in rat striatum after circling training during development.. Brain Res.

[40] Soiza-Reilly M, Fossati M, Ibarra GR, Azcurra JM (2004). Different dopamine D1 and D2 receptors expression after motor activity in the striatal critical period.. Brain Res.

[41] Morris R (1984). Developments of a water-maze procedure for studying spatial learning in the rat.. Journal of Neuroscience Methods.

[42] Fagiolini M, Hensch TK (2000). Inhibitory threshold for critical-period activation in primary visual cortex.. Nature.

[43] Fox K (1992). A critical period for experience-dependent synaptic plasticity in rat barrel cortex.. J Neurosci.

[44] Nisenbaum LK, Webster SM, Chang SL, McQueeney KD, LoTurco JJ (1998). Early patterning of prelimbic cortical axons to the striatal patch compartment in the neonatal mouse.. Dev Neurosci.

[45] Voorn P, Kalsbeek A, Jorritsma-Byham B, Groenewegen HJ (1988). The pre- and postnatal development of the dopaminergic cell groups in the ventral mesencephalon and the dopaminergic innervation of the striatum of the rat.. Neuroscience.

[46] Schapiro S, Salas M, Vukovich K (1970). Hormonal effects on ontogeny of swimming ability in the rat: assessment of central nervous system development.. Science.

[47] St Omer VEV, Mohammad FK (1987). Ontogeny of swimming behavior and brain catecholamine turnover in rats prenatally exposed to a mixture of 2,4-dichlorophenoxyacetic and 2,4,5-trichlorophenoxyacetic acids.. Neuropharmacology.

[48] Bolivar VJ, Manley K, Fentress JC (1996). The development of swimming behavior in the neurological mutant weaver mouse.. Dev Psychobiol.

[49] Schapiro S, Vukovich KR (1970). Early experience effects upon cortical dendrites: a proposed model for development.. Science.

[50] St Omer VE, Bolon B (1990). Secalonic acid D mycotoxin affects ontogeny of brain catecholamines and swimming in mice.. Neurotoxicol Teratol.

[51] Jamon M, Clarac F (1998). Early walking in the neonatal rat: a kinematic study.. Behav Neurosci.

[52] Bruckner G, Grosche J, Schmidt S, Hartig W, Margolis RU (2000). Postnatal development of perineuronal nets in wild-type mice and in a mutant deficient in tenascin-R.. J Comp Neurol.

[53] Brenes JC, Padilla M, Fornaguera J (2009). A detailed analysis of open-field habituation and behavioral and neurochemical antidepressant-like effects in postweaning enriched rats.. Behav Brain Res.

[54] Brenes JC, Rodriguez O, Fornaguera J (2008). Differential effect of environment enrichment and social isolation on depressive-like behavior, spontaneous activity and serotonin and norepinephrine concentration in prefrontal cortex and ventral striatum.. Pharmacol Biochem Behav.

[55] Rossi HL, Neubert JK (2008). Effects of environmental enrichment on thermal sensitivity in an operant orofacial pain assay.. Behavioural Brain Research.

[56] Widman DR, Abrahamsen GC, Rosellini RA (1992). Environmental enrichment: the influences of restricted daily exposure and subsequent exposure to uncontrollable stress.. Physiology & Behavior.

[57] Zaharia MD, Kulczycki J, Shanks N, Meaney MJ, Anisman H (1996). The effects of early postnatal stimulation on Morris water-maze acquisition in adult mice: genetic and maternal factors.. Psychopharmacology (Berl).

[58] Pistell PJ, Nelson CM, Miller MG, Spangler EL, Ingram DK (2009). Striatal lesions interfere with acquisition of a complex maze task in rats.. Behav Brain Res.

[59] Devan BD, McDonald RJ, White NM (1999). Effects of medial and lateral caudate-putamen lesions on place- and cue-guided behaviors in the water maze: relation to thigmotaxis.. Behav Brain Res.

[60] Devan BD, Goad EH, Petri HL (1996). Dissociation of hippocampal and striatal contributions to spatial navigation in the water maze.. Neurobiol Learn Mem.

[61] Francis DD, Zaharia MD, Shanks N, Anisman H (1995). Stress-induced disturbances in Morris water-maze performance: interstrain variability.. Physiol Behav.

[62] Jamon M, Maloum I, Riviere G, Bruguerolle B (2002). Air-stepping in neonatal rats: A comparison of L-dopa injection and olfactory stimulation.. Behav Neurosci.

[63] Stehouwer DJ, McCrea AE, Van Hartesveldt C (1994). L-dopa-induced air-stepping in preweanling rats. II. Kinematic analyses.. Brain Res Dev Brain Res.

[64] Ariano MA, Wang J, Noblett KL, Larson ER, Sibley DR (1997). Cellular distribution of the rat D4 dopamine receptor protein in the CNS using anti-receptor antisera.. Brain Res.

[65] Rubinstein M, Phillips TJ, Bunzow JR, Falzone TL, Dziewczapolski G (1997). Mice lacking dopamine D4 receptors are supersensitive to ethanol, cocaine, and methamphetamine.. Cell.

[66] Kikusui T, Mori Y (2009). Behavioural and neurochemical consequences of early weaning in rodents.. J Neuroendocrinol.

[67] Whitaker J, Moy SS, Saville BR, Godfrey V, Nielsen J (2007). The effect of cage size on reproductive performance and behavior of C57BL/6 mice.. Lab Anim (NY).

[68] Zhu SW, Yee BK, Nyffeler M, Winblad B, Feldon J (2006). Influence of differential housing on emotional behaviour and neurotrophin levels in mice.. Behav Brain Res.

[69] Liu D, Diorio J, Tannenbaum B, Caldji C, Francis D (1997). Maternal care, hippocampal glucocorticoid receptors, and hypothalamic-pituitary-adrenal responses to stress.. Science.

[70] Hartig W, Brauer K, Bruckner G (1992). Wisteria floribunda agglutinin-labelled nets surround parvalbumin-containing neurons.. Neuroreport.

[71] Bennett BD, Bolam JP (1994). Synaptic input and output of parvalbumin-immunoreactive neurons in the neostriatum of the rat.. Neuroscience.

[72] Bracci E, Centonze D, Bernardi G, Calabresi P (2002). Dopamine excites fast-spiking interneurons in the striatum.. J Neurophysiol.

[73] Centonze D, Grande C, Usiello A, Gubellini P, Erbs E (2003). Receptor subtypes involved in the presynaptic and postsynaptic actions of dopamine on striatal interneurons.. J Neurosci.

[74] Sugiyama S, Di Nardo AA, Aizawa S, Matsuo I, Volovitch M (2008). Experience-dependent transfer of Otx2 homeoprotein into the visual cortex activates postnatal plasticity.. Cell.

[75] Huang ZJ, Kirkwood A, Pizzorusso T, Porciatti V, Morales B (1999). BDNF regulates the maturation of inhibition and the critical period of plasticity in mouse visual cortex.. Cell.

[76] Leamey CA, Van Wart A, Sur M (2009). Intrinsic patterning and experience-dependent mechanisms that generate eye-specific projections and binocular circuits in the visual pathway.. Curr Opin Neurobiol.

[77] Liu D, Diorio J, Day JC, Francis DD, Meaney MJ (2000). Maternal care, hippocampal synaptogenesis and cognitive development in rats.. Nature Neuroscience.

[78] Arai JA, Li S, Hartley DM, Feig LA (2009). Transgenerational rescue of a genetic defect in long-term potentiation and memory formation by juvenile enrichment.. J Neurosci.

[79] Gordon JA, Stryker MP (1996). Experience-dependent plasticity of binocular responses in the primary visual cortex of the mouse.. J Neurosci.

[80] Smith SL, Trachtenberg JT (2007). Experience-dependent binocular competition in the visual cortex begins at eye opening.. Nat Neurosci.

[81] Bezard E, Dovero S, Belin D, Duconger S, Jackson-Lewis V (2003). Enriched environment confers resistance to 1-methyl-4-phenyl-1,2,3,6-tetrahydropyridine and cocaine: involvement of dopamine transporter and trophic factors.. Journal of Neuroscience.

[82] van Dellen A, Blakemore C, Deacon R, York D, Hannan AJ (2000). Delaying the onset of Huntington's in mice.. Nature.

